# Characterizing Different Strategies for Resolving Approach-Avoidance Conflict

**DOI:** 10.3389/fnins.2021.608922

**Published:** 2021-02-25

**Authors:** Hector Bravo-Rivera, Patricia Rubio Arzola, Albit Caban-Murillo, Adriana N. Vélez-Avilés, Shantée N. Ayala-Rosario, Gregory J. Quirk

**Affiliations:** ^1^Department of Psychiatry, University of Puerto Rico School of Medicine, San Juan, Puerto Rico; ^2^Department of Anatomy & Neurobiology, University of Puerto Rico School of Medicine, San Juan, Puerto Rico

**Keywords:** individual differences, PVT, accumbens, amygdala, prefrontal

## Abstract

The ability of animals to maximize benefits and minimize costs during approach-avoidance conflicts is an important evolutionary tool, but little is known about the emergence of specific strategies for conflict resolution. Accordingly, we developed a simple approach-avoidance conflict task in rats that pits the motivation to press a lever for sucrose against the motivation to step onto a distant platform to avoid a footshock delivered at the end of a 30 s tone (sucrose is available only during the tone). Rats received conflict training for 16 days to give them a chance to optimize their strategy by learning to properly time the expression of both behaviors across the tone. Rats unexpectedly separated into three distinct subgroups: those pressing early in the tone and avoiding later (Timers, 49%); those avoiding throughout the tone (Avoidance-preferring, 32%); and those pressing throughout the tone (Approach-preferring, 19%). The immediate early gene cFos revealed that Timers showed increased activity in the ventral striatum and midline thalamus relative to the other two subgroups, Avoidance-preferring rats showed increased activity in the amygdala, and Approach-preferring rats showed decreased activity in the prefrontal cortex. This pattern is consistent with low fear and high behavioral flexibility in Timers, suggesting the potential of this task to reveal the neural mechanisms of conflict resolution.

## Introduction

Survival in the wild requires the ability to forage for food while avoiding threats. These behaviors can conflict with each other because threats often co-occur with food availability. When animals are repeatedly exposed to this type of approach-avoidance conflict, they develop strategies to maximize benefits while minimizing costs, but little is known about the development of such strategies. In most studies of approach-avoidance conflict, rodents are required to choose between appetitive and defensive responses to cues that were either conditioned (Vogel et al., 1971; Ramirez-Lugo et al., 2016; Burgos-Robles et al., 2017; Hamel et al., 2017; Schumacher et al., 2018; Choi et al., 2019; Oberrauch et al., 2019) or innate (Choi and Kim, 2010; Dopfel et al., 2019). These studies required rats to make a single “either/or” decision in a given trial, and were not designed to allow rats to achieve both approach and avoidance outcomes within a trial. Furthermore, rats were not given a period of conflict training sufficiently long enough to allow for the development of strategies to maximize reward without compromising avoidance. The development of conflict strategies has been studied in non-aversive conflict tasks, such as effort-based decision-making (less work, small reward vs. more work, large reward) (Winstanley et al., 2004; Floresco and Magyar, 2006; Floresco and Ghods-Sharifi, 2007; Floresco et al., 2008b; Eubig et al., 2014), delayed discounting (rapid small reward vs. delayed large reward) (Winstanley et al., 2004; Floresco et al., 2008a; Floresco and Whelan, 2009; Ghods-Sharifi and Floresco, 2010), and probabilistic discounting (certain small reward vs. uncertain large reward) (Bjork et al., 2007; St Onge et al., 2011; Montes et al., 2015). These studies have shown that rodents are capable of developing strategies for maximizing rewards and minimizing loss; however, less has been studied regarding such strategies under threatening situations.

Previous studies of approach-avoidance conflict have shown that animals prioritize avoidance over approach responses, as evidenced by the suppression of reward-seeking behavior in the presence of threats (Muenzinger, 1936; Maslow, 1943; Bravo-Rivera et al., 2014, 2015; Jean-Richard-Dit-Bressel and McNally, 2015; Burgos-Robles et al., 2017; Martinez-Rivera et al., 2019; Capuzzo and Floresco, 2020; Sutton and Krashes, 2020). To study the development of strategies in such situations, we designed a simple approach-avoidance task that pits the motivation to forage for sucrose pellets against the motivation to avoid a footshock. During a 30 s tone that signals both the availability of food and the occurrence of a 2 s shock at the end of the tone, rats could either press a bar for sucrose pellets or step onto a safe platform located far from the bar (Bravo-Rivera et al., 2014). This design allowed animals to optimize their conflict strategy by pressing the bar during the early portion of the tone while postponing avoidance until the latter portion of the tone. We administered conflict training for 16 days (9 trials per day) to allow sufficient time for trial-and-error learning and strategy development. We correlated individual differences in rats’ chosen strategies with neural activity in several structures, using cFos immunohistochemistry. We focused on the medial prefrontal cortex, the nucleus accumbens (NAcc), and the basolateral amygdala (BLA), areas implicated in both approach (Ambroggi et al., 2008; Stuber et al., 2011; Burgos-Robles et al., 2013; Beyeler et al., 2016; Piantadosi et al., 2020) and avoidance (Martinez et al., 2013; Moscarello and LeDoux, 2013; Bravo-Rivera et al., 2014, 2015; Ramirez et al., 2015) behavior.

## Materials and Methods

All experimental procedures were approved by the Institutional Animal Care and Use Committee at the University of Puerto Rico School of Medicine. A total of 69 male and 36 female Sprague-Dawley rats (aged between 3 and 5 months) were used in these experiments. Rats weighed 300–350 g and were food deprived to 85% of their body weight at the start of avoidance training. All rats had *ad libitum* access to water. Rats were housed in individual cages a 12:12 light cycle with tests occurring in the light phase to facilitate comparison with prior studies from this lab.

### Avoidance Training

**FIGURE 1:**
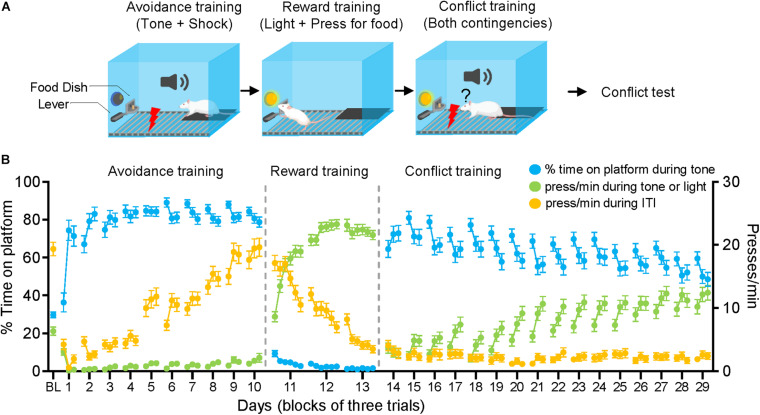
Approach-avoidance conflict training. **(A)** Rats were given 10 days of avoidance conditioning followed by 3 days of reward conditioning and 16 days of conflict training. **(B)** As rats learned avoidance conditioning, they increased the time spent on the platform (blue line), while reducing pressing during the tone (relative to the baseline established during the first tone) (green line). The pressing during the intertrial interval (ITI) (ITI = 1 min prior to each trial) (orange line) returned to preconditioning baseline (BL) by the end of avoidance conditioning. During reward conditioning, rats decreased their pressing during the ITI and increased their pressing during the light (green line). When the tone-light were co-presented in conflict training, rats gradually reduced the time spent on platform and gradually increased their pressing.

All rats were previously trained to press a lever for sucrose pellets at a variable interval feeding schedule of 30 s (VI30) prior to platform mediated avoidance (PMA) training. Rats were conditioned and tested in the same operant chambers used for lever press training (26.67 cm, long, 27.94 cm wide, 27.94 cm tall, Coulbourn Instruments, located in sound-attenuating cubicles). The floors consisted of stainless-steel rods that delivered scrambled foot shocks (see Figure 1). Rats were conditioned with a pure tone (30 s, 4 kHz, 75 dB) paired with a co-terminating shock (2 s, 0.4 mA). Inter-trial intervals were variable, averaging 3 min. Sucrose pellets were continuously available via a lever-pressing at a variable interval schedule of reinforcement averaging 30 s (VI-30). An acrylic platform (13.97 cm on each side, 0.33 cm tall) was placed in the corner opposite from the sucrose lever where rats could step on to avoid the footshock. PMA training occurred over 10 days, with 9 trials per day.

### Reward Training

After completing avoidance training, rats were placed in the same operant chambers with sucrose pellets available only during 30 s periods when a light cue positioned above the pellet dispenser was illuminated (see Figure 1). A pellet was dispensed with each lever press (one-to-one). Each reward conditioning session consisted of 20 trials and ∼180 s inter-trial intervals. A total of three sessions were given across 3 days, a point where most animals reached the criterion of limiting pressing to the period of the light cue.

### Conflict Training

Conflict training started 24 h after completing reward training, within the same operant chambers (see Figure 1). As during reward training, sucrose pellets were available only during the 30 s period in which the light cue signaled food availability. Shocks co-terminated with the 30 s tone as during avoidance training. However, unlike previous phases, the tone and light were co-presented during each trial for 30 s. Each day, a total of 9 trials of tone-light co-presentations were delivered with inter-trial intervals of ∼180 s. Conflict training was continued for 16 days to allow rats to adopt a stable behavioral strategy (see Figure 1B).

### Conflict Test

The Conflict test took place 24 h after day 16 of conflict training. Rats were exposed to three tone-light trials during the conflict test session. These trials were presented without shock, and sucrose pellets were delivered under the same conditions of conflict training. The behavior in the first trial was used to separate the rats into different groups and define the phenotypes. This was done to avoid any effects of extinction that might be observed in the subsequent shock-free trials.

### Open Field Test

Forty-eight hours after the conflict test, rats were placed in an all-black, circular open field (90 cm in diameter and 50 cm tall walls). We designated the outermost circular section of the open field (12.7 cm in width) as the periphery. This section occupied an area of 3,084 cm^2^ (48% of the total arena area). The rest of the area (52%) was considered the center region.

### Social Exploration Test

After the open field test, we immediately placed a circular wire-mesh cage (30 cm in diameter and 45 cm tall) in the center of the open field. Rats were allowed 3 min to explore around this cage. Then an unfamiliar conspecific of the same sex was placed inside the cage for an additional 3 min, and the rat could continue to explore the area around the cage. The ring area around the cage (12.7 cm in width) occupied 1,704 cm^2^ (30% of the total available area). Social interaction was measured as the % time spent in this ring area across the 3 min.

### Data Collection and Analysis

All behavior was recorded with digital video cameras. Commercially available software (AnyMaze, Stoelting) was used to assess freezing, time on the platform, and social interaction time. Trials were averaged in blocks of three and compared with a Student’s *t*-tests (two-tailed) or ANOVA followed by Tukey *post-hoc* tests (Prism; GraphPad).

To quantify approach and avoidance behaviors, we compared the % time rats spent on the platform with their average press rate (presses/min) across the 30 s tone. Presses were rewarded on a VI-30 schedule during avoidance training and on a one-to-one schedule (during the CS) in reward training, conflict training, and conflict test.

### Immunocytochemistry

Ninety minutes after conflict test, animals were anesthetized with sodium pentobarbital (450 mg/kg, i.e.) and perfused transcardially with 250 mL of saline (0.9%), followed by 750 mL of 4% (vol/vol) paraformaldehyde in 0.1 phosphate buffer (pH 7.4). Brains were post-fixed for 3 h in the same fixative solution and transferred to a 30% sucrose solution in a 0.1 M phosphate buffer at 4°C for 48 h. Brains were frozen, and a series of 40-μm sections were cut with a cryostat (CM 1850; Leica) along the frontal plane and collected at different coronal levels from the mPFC to the amygdala. Briefly, sections for the mPFC, BLA, and VS were initially blocked in a solution of 2% normal goat serum (NGS; Vector Laboratories, Burlingame, CA) plus 0.3% triton (Triton X-100; St. Louis, MO) in 0.12 M potassium buffer saline for 1 h and then incubated overnight at room temperature with rabbit anti-cFos antibody (1:500; ABE457, EMD Millipore). 24 h later, slices were incubated with anti-rabbit biotinylated secondary antibody (1:200, Vector Laboratories) for 2 h and placed in the mixed avidin-biotin horseradish peroxidase complex solution (1:200; ABC Elite kit, Vector Laboratories) for 90 min. Black immunoreactive nuclei labeled for cFos were visualized after ∼10 min of exposure to a chromogen solution containing 0.02% 3,39 diaminobenzidine tetrahydrochloride with 0.3% nickel ammonium sulfate (DABNi) in 0.05 M Tris buffer (pH 7.6). Slices were then mounted and coverslipped.

Image thresholding and cell counts were performed blind with respect to group assignment. The identities of the rats were only revealed after all counts were completed. Images for infralimbic cortex (IL +3.00 to +3.52 AP), prelimbic cortex (PL +3.00 to +3.52 AP), basolateral amygdala (BLA −3.00 to −2.00 AP), the lateral portion of the central amygdala (CeL −3.00 to −2.00 AP), medial portion of the central amygdala (CeM −3.00 to −2.00 AP), paraventricular thalamus (PVT −3.00 to −2.00 AP), nucleus accumbens core (NAcC +2.00 to 0.00 AP), and, nucleus accumbens shell (NAcSh +2.00 to 0.00 AP), were digitized with a microscope (Model BX51, Olympus, Tokyo, Japan) at 20× magnification using a digital camera (Model DP72, Olympus, Tokyo, Japan). For the NAcC, NAcSh, BLA, CeL, CeM, and PVT, pictures were taken and stitched together using commercially available software (Image Composite Editor, Microsoft). Then, the whole structure was delineated in the stitched picture using adjacent Nissl-stained slices. cFos positive cells were automatically counted using software (Metamorph, 6.1). The density of cFos labeled cells was calculated by dividing the number of cFos labeled cells by the area of the counted region. The cFos densities for each structure were measured in both hemispheres at three different sections and averaged to produce the final value used for each rat. Structures that showed group differences were reported in Figures 4B–D and structures that showed no difference are reported in Supplementary Figure 2A.

## Results

Sixty-nine male rats were given approach-avoidance conflict training in three stages: avoidance training, reward training, and conflict training (Figure 1A). During avoidance training (10 days), rats increased the time spent on the platform while decreasing their press rate during the tone (Figure 1B). During the intertrial interval (ITI), pressing dropped initially but gradually returned to pre-conditioning levels as rats likely learned that shocks never occurred during the ITI. In the reward training phase (3 days), the sucrose availability was restricted to 30 s periods signaled by a light-cue above the lever (no tones or shocks were delivered). Rats increased their press rate during the light-cue and decreased their press rate during ITIs (Figure 1B). During conflict training (16 days), the tone and light cues were co-presented for 30 s, requiring rats to balance pressing vs. avoiding during the tone-light period. Twenty-four hours after conflict training, rats were given three tone-light trials (without a shock), to assess their conflict strategy.

### Conflict Training Resulted in Distinct Strategies

**FIGURE 2:**
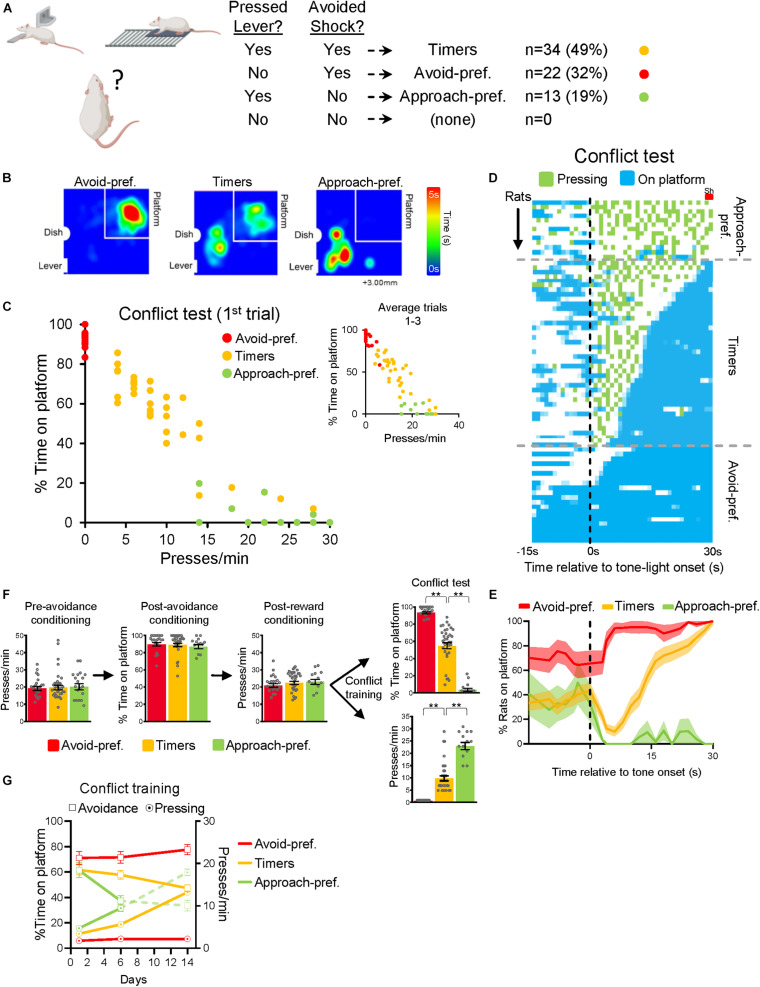
Rats showed three distinct strategies for resolving approach-avoidance conflict. **(A)** Criteria for separating rats into different conflict strategies. Rats were separated into three subgroups: Avoidance-preferring (22/69 32%, red), Approach-preferring (13/69, 19%, green), and Timers (34/69, 49%, yellow). **(B)** Heat maps showing the location of rats during the conflict test (*n* = 7 for each subgroup). **(C)** Presses/min vs. % time pressing for individual rats at conflict test trial 1, showing that the three subgroups were distinct from each other. Inset shows averaged data for all three trials of conflict test. **(D)** Actogram depicting each rat’s behavior during conflict test (bin = 1 s) across the conflict test. Subgroups are separated by horizontal dashed lines. **(E)** Average time spent on platform for each subgroup. **(F)** Averaged behaviors for each subgroup prior to, and following, conflict training. Subgroup differences were not apparent prior to conflict training. **(G)** Average time spent avoiding (square) and pressing (circle) during conflict training, for each subgroup. **P < 0.01.

Rats initiated conflict training with high levels of avoidance and low levels of pressing (Figure 1B). As conflict training progressed, rats gradually reduced their time avoiding and gradually increased their rate of pressing. However, behavioral responses were quite variable across rats. We therefore divided the rats into separate subgroups based on their behavior. Because the two goals of the task were to press for sucrose and/or avoid shock, we classified rats based on whether or not they achieved these goals during the first trial of the test session (see table in Figure 2A). Rats that successfully avoided the shock (positioned on the platform during seconds 28–30) and pressed for sucrose (pressing at least once during the tone) constituted 49% of rats (*n* = 34). Rats that successfully avoided the shock but never pressed for sucrose constituted 22% (*n* = 22). Rats that pressed for sucrose but failed to successfully avoid the shock constituted 19% (*n* = 13). Finally, rats that neither successfully avoided nor pressed were not observed (0%). We termed the group that both pressed and avoided as “Timers,” the group that avoided but did not press as “Avoidance-preferring,” and the group that pressed but did not avoid as “Approach-preferring.” We based this classification on the first trial of the test only so as to avoid any effects of extinction that might occur in the subsequent trials.

*Post-hoc* analysis showed that Approach-pref. rats received more shocks by the end of conflict training than the other two subgroups (41% of trials compared to 9% in Timers and 5% in Avoid-pref. rats [one-way ANOVA: *F*_(2, 66)_ = 58.99; *p* < 0.001, Tukey *post-hoc*: *p* < 0.001]. Avoid-pref. rats spent most of their time on the platform, Approach-pref. rats spent most of their time near the lever, and Timers divided their time between these locations (Figure 2B, see Supplementary Video). A comparison of the time on platform vs. press rate showed these subgroups fell largely into separate clusters, with the exception of Timers, which divided into high-pressing and low-pressing subgroups (Figure 2C). A similar distribution of subgroups was found in female rats (Supplementary Figure 1). In the second and third test trials, some rats increased their press rate and shifted to the right, as evidenced in the inset of Figure 2C.

Figure 2D shows time spent pressing or avoiding for each second of the tone-light cue at test for each rat. The Timer subgroup limited pressing mostly to the first half of the 30 s tone-light cue, but the latency to initiate avoidance varied considerably from 5 to 28 s (shocks occurred during seconds 28–30). Figure 2E shows that, on average, Avoid-pref. remained on the platform, Approach-pref. never moved to the platform, but Timers gradually increased their time on the platform across the tone-light stimulus.

Interestingly, no subgroup differences were detected prior to conflict training (Figure 2F). All subgroups showed similar: pressing at the start of the experiment [*F*_(2, 66)_ = 0.0123; *p* = 0.98]; time on platform during avoidance training [*F*_(2, 66)_ = 0.299; *p* = 0.74]; pressing at the end of reward training [*F*_(2, 66)_ = 0.212; *p* = 0.81]; and shock sensitivity (measured by comparing subgroup’s maximum speed of running during the first shock exposure [*F*_(2, 52)_ = 0.169; *p* = 0.84] (Supplementary Figure 3A). Early in conflict training, the subgroups behaved similarly and then diverged as training progressed (Figure 2G). In the Timer and Approach-pref. subgroups, avoidance gradually diminished as press rates increased; however, the time spent in each of these behaviors converged at different time points (day 6 for the Approach-pref. and day 14 for the Timers subgroup). In contrast, the Avoid-pref. subgroup showed no such convergence, exhibiting high levels of avoidance and low press rates throughout conflict training. Excessive avoidance may be driven by increased fear, as Avoid-pref. rats showed elevated freezing levels by the end of conflict training [*F*_(2, 57)_ = 10.40; *p* < 0.001, Tukey *post-hoc*: *p* = 0.046, *p* = 0.002] (Supplementary Figure 3B). Thus, conflict training triggered the emergence of different strategies in these subgroups.

### Approach-Preferring Rats Showed Reduced Social Exploration

**FIGURE 3:**
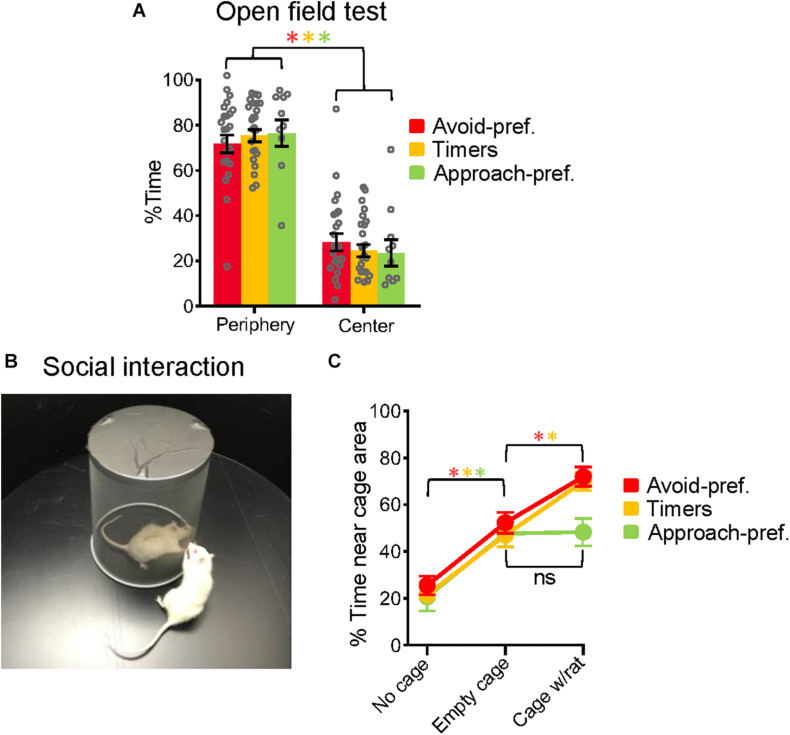
Approach-preferring rats showed reduced social exploration. **(A)** The three subgroups showed no differences in the % of time exploring the center of the open field, a measure of innate fear. **(B)** Experimental setup for the exploration task in which rats could explore the outside of a novel cage that was first empty and later contained a demonstrator rat. **(C)** Placing the empty cage in the center of the open field increased the time spent exploring near the cage in all three subgroups. Adding a demonstrator rat to the empty cage further increased the time spent exploring for Avoid-pref. and Timer subgroups, but not in the Approach-pref. subgroup. Avoid-pref., *n* = 23; Timers, *n* = 24; Approach-pref., *n* = 10 (Student’s *t*-tests *p < 0.05, Bonferroni corrected).

To determine if the subgroups showed behavioral differences apart from conflict phenotypes, we used a circular open field to evaluate anxiety and social exploration. Consistent with previous findings (Denenberg, 1969), all three subgroups avoided the center of the open field with no significant differences in the percent time spent in the center [Avoid-Pref.: 28%, Timers: 25%, Approach-pref.: 24%, *F*_(2, 54)_ = 0.414, *p* = 0.66] (Figure 3A). After 3 m, a novel wire-mesh cage was placed in the center, and the three subgroups spent much of their time near this cage (Avoid-Pref.: from 28 to 52%, Timers: 25–47%, Approach-pref.: 24–48%, all t’s > 3.54, *p* < 0.05, Bonferroni corrected) (Figure 3B). After an additional 3 m, a same-sex demonstrator rat was placed inside the wire mesh cage, to assess rats’ social exploration. This further increased the time rats spent near the cage in Avoid-Pref. (52–72%) and Timers (57–70%) subgroups (Avoid-Pref.: *t*_22_ = 3.10; Timers: *t*_23_ = 4.14, *p* < 0.05, Bonferroni corrected), but not in Approach-pref. rats (48–48%, *t*_9_ = 0.11, *p* = 0.91) (Figure 3C). Thus, despite similar levels of anxiety and novel object exploration, Approach-pref. rats showed reduced social exploration relative to the other subgroups. While these findings suggest that differences in anxiety levels may not contribute to subgroup differences, a final answer to this question would require assessing the effects of anxiolytic drugs on each subgroup in the conflict task.

**FIGURE 4:**
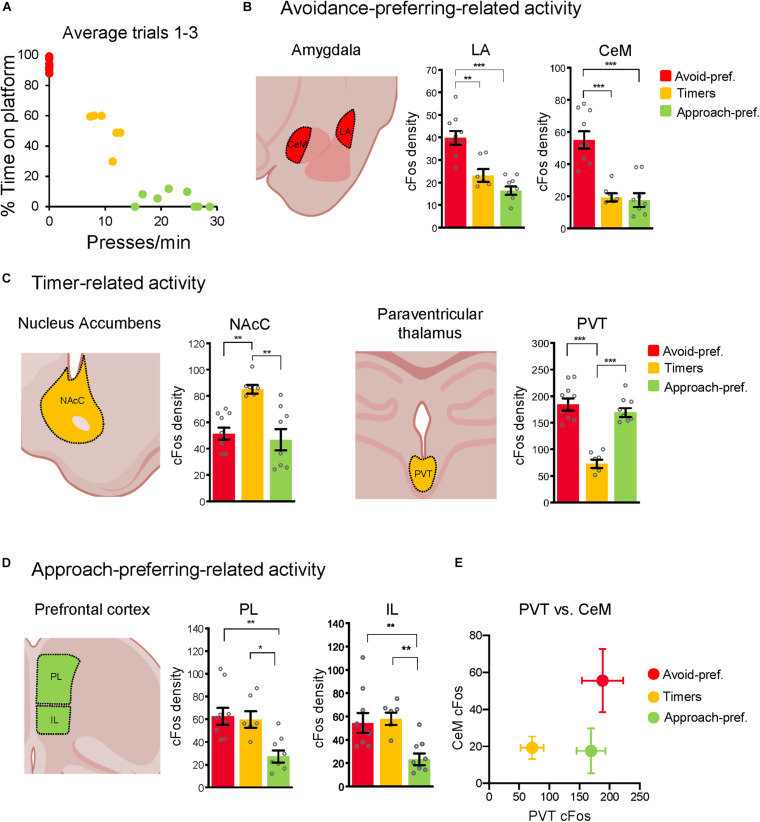
The three subgroups showed distinct neuronal activity patterns. **(A)** Average presses/min vs. % time on platform for the three trials at conflict test in rats included in the cFos analysis. Only rats with consistent subgroup behavior were selected for cFos analysis. **(B)** Rats were euthanized 90 min after the conflict test and processed for cFos. Avoid-pref. Rats showed elevated activity in the lateral (LA) and centromedial (CeM) subregions of amygdala, relative to the other two subgroups. **(C)** Timer rats showed elevated activity in the nucleus accumbens-core (NAcC), and reduced activity in the paraventricularthalamus (PVT), relative to the other two groups. **(D)** Approach-pref. rats showed reduced activity in the prelimbic (PL) and infralimbic (IL) subregions of medial prefrontal cortex, relative to the other two subgroups (one-way ANOVA*p < 0.05, **p < 0.01, ***p < 0.001 Tukey *post-hoc* test). **(E)** Activity within CeM and PVT separated the three subgroups, consistent with low levels of fear and high levels of behavioral flexibility in Timer rats.

### Neuronal Activity Profiles in the Three Subgroups

The animals used in our cFos analysis were selected to exhibit the three distinct subgroups stably across each of the three trials (behavior shown in Figure 4A). Rats were euthanized 90 m following the conflict test, and cFos-labeled neurons were counted blind with respect to behavior (Beck and Fibiger, 1995; Navarro et al., 2000; Supplementary Figure 2A). The Avoid-pref. subgroup showed increased activity in the lateral amygdala (LA) [*F*_(2, 20)_ = 22.52; *p* < 0.001, Tukey *post-hoc* test: *p* = 0.001] and medial division of the central nucleus of amygdala (CeM) [*F*_(2, 20)_ = 22.28; *p* < 0.001, Tukey *post-hoc* test: *p* < 0.001] relative to the other two subgroups (Figure 4B and Supplementary Figure 2B for structures that did not show group differences). This is consistent with previous studies linking the activation of these areas with conditioned fear expression (Pare et al., 2004; Namburi et al., 2015). The Timers showed increased activity in the nucleus accumbens core (NAcC), [*F*_(2, 20)_ = 10.47; *p* < 0.001, *post-hoc* test: both *p*’s < 0.003] (Figure 4C), as well as decreased activity in the paraventricular nucleus of the thalamus (PVT) relative to the other subgroups [*F*_(2, 20)_ = 32.52; *p* < 0.001, *post-hoc* tests: both *p*’s < 0.001]. The Approach-pref. subgroup showed decreased activity in prelimbic (PL) cortex [*F*_(2, 20)_ = 7.42; *p* = 0.003, *post-hoc* test: both *p*’s < 0.009], and infralimbic (IL) cortex [*F*_(2, 20)_ = 8.51; *p* = 0.002, *post-hoc* test: both *p*’s < 0.012] relative to the other two subgroups (Figure 4D), consistent with the necessity of the medial prefrontal cortex for expression of active avoidance (Moscarello and LeDoux, 2013; Bravo-Rivera et al., 2014, 2015; Capuzzo and Floresco, 2020). Combining PVT and CeM expression levels revealed distinct activity profiles for each subgroup (Figure 4E).

## Discussion

We developed a rodent task that pits pressing for sucrose against avoidance of shock to study approach/avoidance conflict resolution. Rats were separated into three distinct subgroups based on their acquired strategies: those preferring to approach, those preferring to avoid, and those capable of balancing both behaviors by taking into account the timing of the shock. All three subgroups showed distinct neuronal activity profiles, suggesting possible circuits involved in resolving approach/avoidance conflicts.

Previous conflict studies have not focused on strategy development in approach-avoidance conflict. Rather, much work has focused on conflicts between riskier large rewards and safer small rewards (Friedman et al., 2015), punished rewards (Alberini, 2005; Ramirez-Lugo et al., 2016; Piantadosi et al., 2017), or the expression of freezing during reward availability. Notably, a recent study featured a novel approach-avoidance conflict task in which food-deprived mice had to choose between low-effort/high-threat rewards and high-effort/no-threat rewards (Oberrauch et al., 2019). However, mice were not able to achieve both goals within a given trial (maximizing food reward while also preventing shock). An important distinction of our task is that rats could combine foraging with avoidance without compromising either goal within a single trial. Furthermore, the extended training we used allowed animals to develop a strategy through trial and error, as observed in the changing behavior across the 16 days of conflict training.

Timing behavior in our task relies on the animals’ internal regulation of both approach and avoidance behaviors. For example, at CS onset Timer rats must suppress the initial urge to avoid to accommodate pressing, and later, they must suppress pressing to accommodate avoidance. Timers achieved this by adapting previously learned behaviors for use under conflict conditions. Because they expressed both pressing and avoidance, increased activity in NAcC in Timers is consistent with the activation of this structure by both foraging (Ambroggi et al., 2008) and avoidance (Bravo-Rivera et al., 2014, 2015). Increased activity in NAcC also concurs with studies showing that NAcC is necessary for the expression of both active avoidance (Bravo-Rivera et al., 2014; Piantadosi et al., 2018) and foraging (Nicola et al., 2005; Ambroggi et al., 2011). Another possibility is that NAcC activity is necessary for the behavioral inhibition that allows Timers to delay avoidance early in the tone-light and to stop pressing to mount the platform. Indeed, inactivation of NAcC has been shown to impair the suppression of avoidance during reward-seeking (Schwartz et al., 2017) and to impair suppression of pressing for a small food reward in favor of a larger reward (Cardinal et al., 2001).

Avoid-pref. rats showed excessive avoidance at the cost of access to reward, consistent with the increased freezing we observed in this subgroup. Increased fear in these animals could impair behavioral flexibility and prevent them from taking the necessary risks for timing. This subgroup seemed to value avoidance of the shock more than the acquisition of food during conflict training, resulting in the loss of potential rewards. Because they expressed increased avoidance and freezing, high activity in LA and CeM is consistent with previous studies linking activation of these areas with fear expression (Pare et al., 2004; Namburi et al., 2015). Consistent with this, prior work has shown that a large portion of BLA-CeM projections originate in LA and that these neurons encode mostly negative valence compared to BLA-NAcc and BLA-VHipp (Beyeler et al., 2016, 2018; Namburi et al., 2016; Kahn et al., 2020). Avoid-pref. rats may resemble anxiety disorder patients who sacrifice rewarding opportunities because of excessive avoidance. Thus, Avoid-pref. rats could serve as a cost-focused model of anxiety disorders, in which excessive drive to avoid prevents the pursuance of rewarding activities (Berenbaum and Connelly, 1993; Berghorst et al., 2013; Dillon et al., 2014).

Timers showed decreased activity in PVT which is consistent with prior studies showing that PVT plays a crucial role in choice selection during approach-avoidance conflict (Choi and McNally, 2017; Choi et al., 2019). In our task, Timers learned to switch from cue-signaled pressing to cue-signaled avoidance, which correlated with the reduction in PVT activity we observed. Other studies have shown that PVT encodes salient features of aversive and rewarding stimuli (Do-Monte et al., 2017; Zhu et al., 2018; Gao et al., 2020). Increased PVT activity could promote excessive salience to the tone or light in the Avoid-pref. or Approach-pref. rats, respectively, thereby preventing them from pursuing multiple goals simultaneously. Reduced PVT activity in Timers may reflect lower salience of both stimuli, thereby enabling controlled expression of both behaviors. If increased PVT activity impairs behavioral flexibility, Timers would be expected to show reduced activity in both CeM and PVT. In fact, a comparison of CeM and PVT activity patterns clearly distinguished the three subgroups (Figure 4E), with Timers showing low activity in both structures and Avoid-pref. showing high activity in both structures. We therefore propose that the Timer subgroup may serve as a model for understanding the role of behavioral inhibition in conflict resolution.

The Approach-pref. subgroup exhibited little to no avoidance and showed low levels of prefrontal activity (both PL and IL). Previous studies demonstrate that PL activity is necessary to express platform-mediated avoidance (Bravo-Rivera et al., 2014, 2015; Diehl et al., 2018), and PL neurons show activation during this type of avoidance (Diehl et al., 2018; Martinez-Rivera et al., 2019). Therefore, it is likely that the reduced activity in PL reflects the lack of avoidance in this subgroup (Bravo-Rivera et al., 2015). Reduced activity in PL could also reflect a lack of behavioral inhibition, as evidenced by this subgroup’s inability to terminate pressing. Hypoactivity in rodent PL was correlated with an inability to terminate drug self-administration under the threat of punishment (Chen et al., 2013; Hu et al., 2019), and inactivation of PL impaired rats’ ability to terminate food-seeking under similar conditions (Verharen et al., 2019). Pursuing reward despite negative consequences is a hallmark of substance abuse disorders (Leshner, 1997), together with impaired social behavior (Fowler et al., 2007). Indeed Approach-pref. rats displayed reduced social interactions, and chemogenetic inhibition of IL-BLA circuitry was recently shown to impair social behaviors (Huang et al., 2020). Thus, the Approach-pref. subgroup may model individuals at elevated risk for addictive disorders.

In summary, we developed a simple rodent task to study the development of approach-avoidance conflict-resolution strategies. We observed three subgroups with distinctive neural activity patterns. While we do not have data on the ventral hippocampus (vHPC), recent studies have shown interesting findings regarding the role of vHPC during approach-avoidance conflict (Ito and Lee, 2016; Schumacher et al., 2016, 2018). Interestingly, distinct regions within the vHPC have been shown to play opposite roles in approach-avoidance conflict (Schumacher et al., 2018). Future studies should address the role of this structure in our task. Furthermore, additional studies could reveal potential genetic or epigenetic factors that could shape the observed behavioral phenotypes and their possible role in anxiety and addiction (Simmons et al., 2012; Flagel et al., 2016; Morrow and Flagel, 2016). Future questions might be: how do these phenotypes develop through early or later experience? How does an animal’s specific thresholds for motivation (e.g., hunger vs. safety) drive its conflict strategy? Such questions would be amenable to a cost-benefit analysis from a neuroeconomic perspective (Corr and McNaughton, 2012; McNaughton and Corr, 2014).

## Data Availability Statement

The raw data supporting the conclusions of this article will be made available by the authors, without undue reservation.

## Ethics Statement

The animal study was reviewed and approved by the Institutional Animal Care and Use Committee of the University of Puerto Rico School of Medicine, Association for Assessment and Accreditation of Laboratory Animal Care (AAALAC).

## Author Contributions

HB-R and GQ: conceptualization, methodology, writing—original draft, writing—review and editing, and supervision. HB-R, PR-A, AC-M, AV-A, and SA-R: investigation. GQ: funding acquisition and resources. All authors contributed to the article and approved the submitted version.

## Conflict of Interest

The authors declare that the research was conducted in the absence of any commercial or financial relationships that could be construed as a potential conflict of interest.
