# Supplementary figures and images for: Characterizing Different Strategies for Resolving Approach-Avoidance Conflict
Source: Front Neurosci. 2021 Feb 25;15:608922. doi: 10.3389/fnins.2021.608922 (PMC7947632; doi:10.3389/fnins.2021.608922)

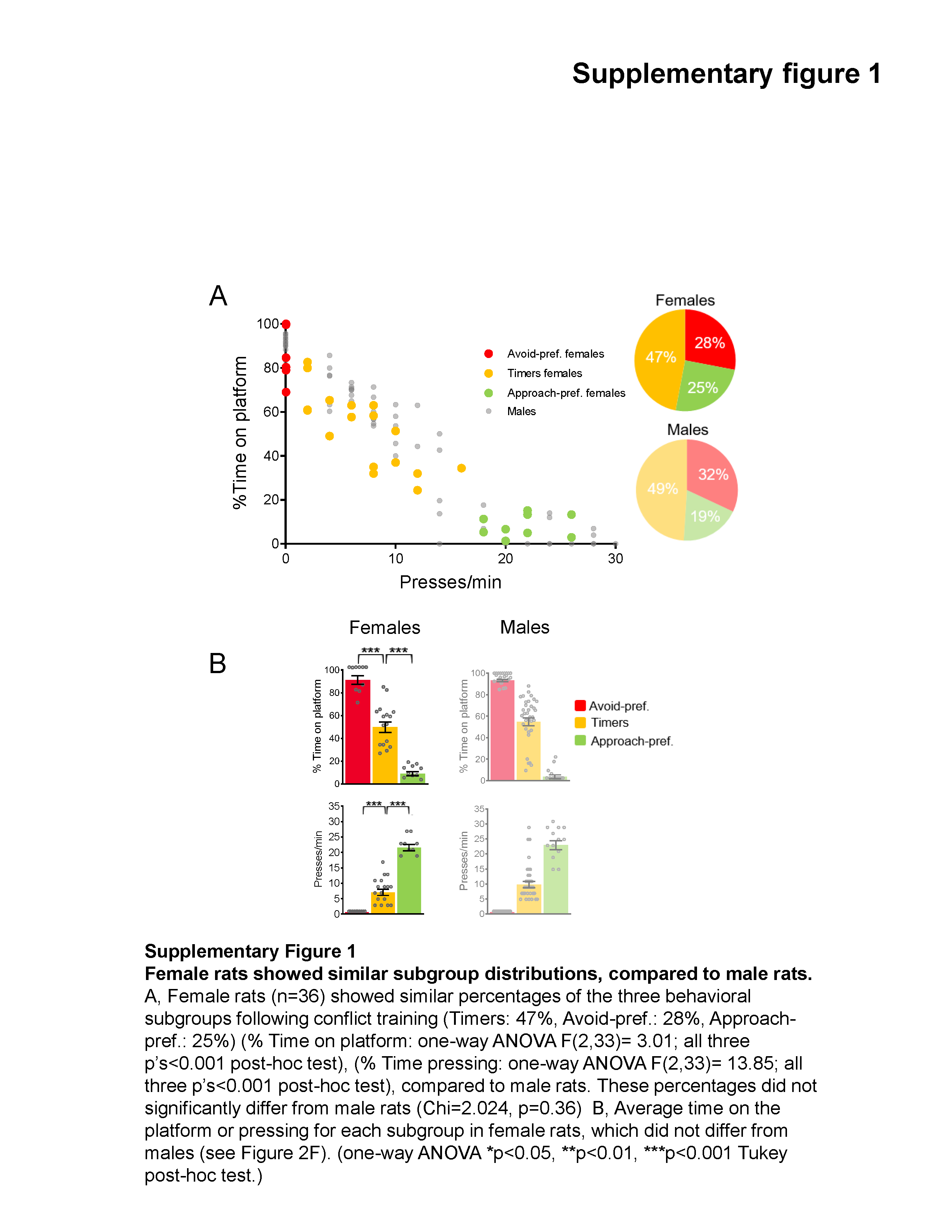

Supplement: Supplementary file 1 [file Image_1.TIF]

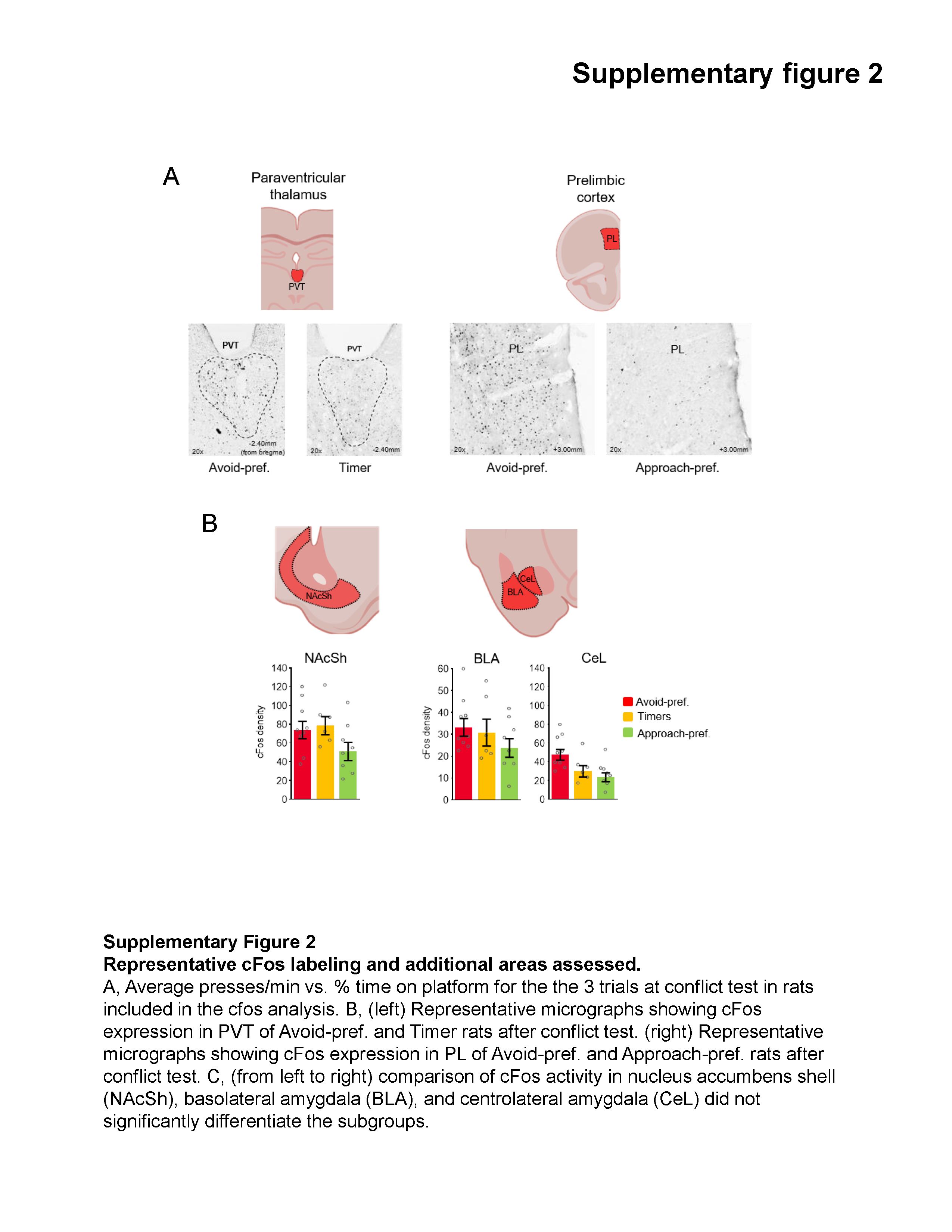

Supplement: Supplementary file 2 [file Image_2.TIF]

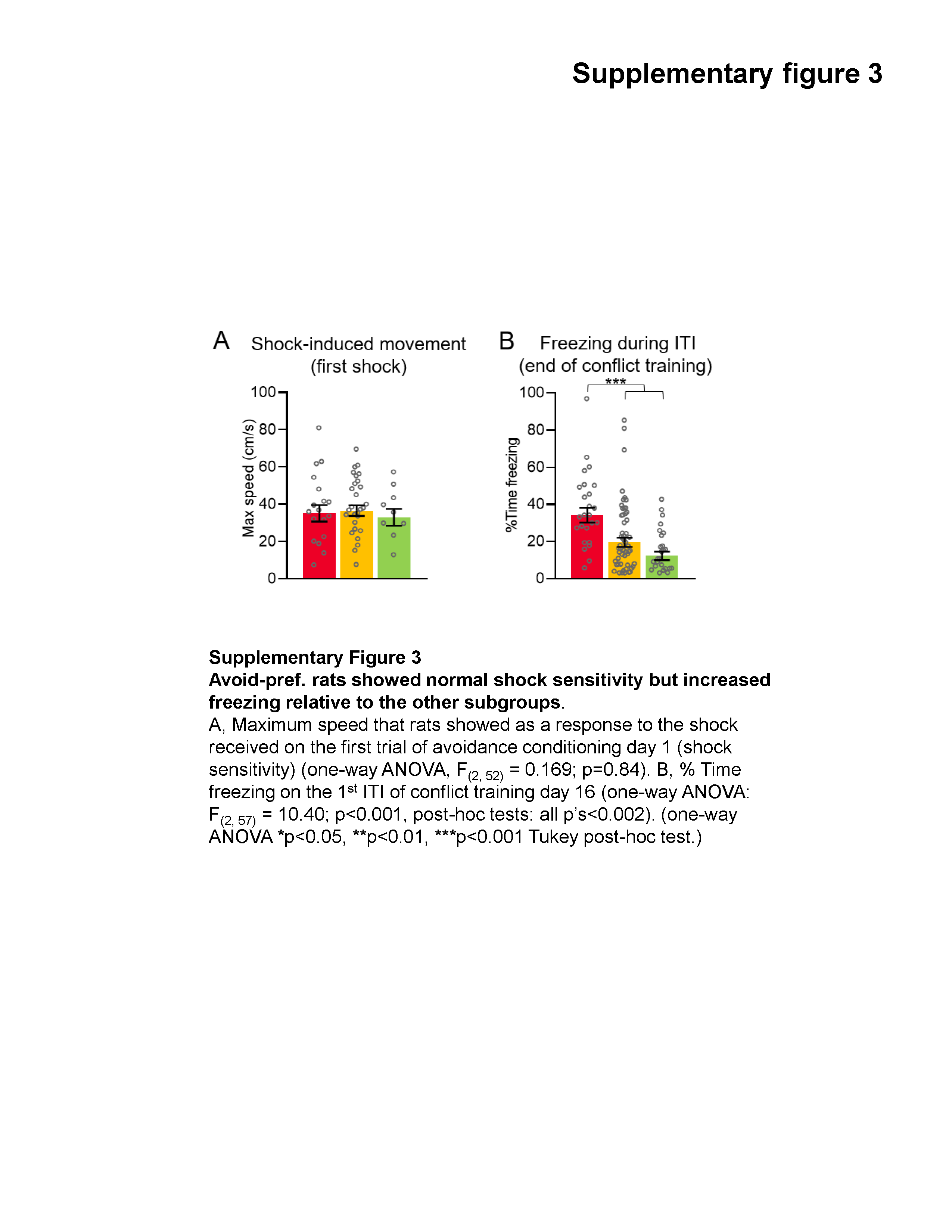

Supplement: Supplementary file 3 [file Image_3.TIF]
